# 

**Published:** 1895-05

**Authors:** 


### AMERICAN JOURNAL

OF

### DENTAL SCIENCE.

17

EDITED BY

F. J. S. GORGAS, M. D., D. D. S.,

RICHARD GRADY, M. D., D. D. S.

VOLUME XXIX—THIRD SERIES.

(1895-6.)

BALTIMORE:
SNOWDEN & COWMAN MFG. CO.

SURGEON GEN'L'S OFFICE
158158.
LIBRARY

LONDON;
TRUBNER & CO,, 60 PATERNOSTER ROW.

1896.

### Index to Vol. XXIX.---Third Series.

A Few More Words on Pyorrhœa ........................ 13
A Case in Practice...... ............................ 37
A Note on Capping a Tooth Pulp....................... 46
An Act to Amend and Re-enact Sections 1767 and 1774 of the Code of Virginia............................ 58
Admission to Study Dentistry......................... 65
American Dental Association ......................... 86
A Praiseworthy Effort: A Reference Library for Dentists.. 87
Aluminum Plates...................................... 88
An Important Decision—The Law Regulating Dentistry in Tennessee Tested................................. 89
American Dentistry in London......................... 90
A New Porcelain...................................... 96
Amalgam—Its Use from a Practical Standpoint.......... 97
Attitude of the Dentist Towards His Patient .......... 124
Another Method of Banding a Logan Crown............... 134
American Dental Association Meeting................... 136
American Dental Association........................... 138
Augustus Woodruff Brown, D. D. S...................... 139
Antral Empyema of Tuberculous Origin.................. 144
A Digest.............................................. 179
Amalgam............................................... 187
Antisepsis in Dentistry............................... 189
Arsenic............................................... 192
Anæsthesia vs Asphyxia................................ 211
Amalgam and How to Manipulate It ..................... 225
American Dental Colleges.............................. 227
A Practical Point in the Use of Disinfectants and Antiseptics, 270
Alveolar Abscess...................................... 276
Argonin............................................... 287
A Vent in Your Port Polisher.......................... 287
Alcoholism in France.................................. 288
A Broken Plate-Holder................................. 332
A Curious Case........................................ 334
Abscesses ............................................ 384
Ancient and Modern Hypnotism.......................... 433
A Case of Complete Separation of the Upper Jaw........ 474
Aluminum Plates....................................... 477
A Compatible Antiseptic............................... 480
A Few Notes on Dental Prosthetics..................... 508
Antisepsis............................................ 512
A New Substitute for Gold............................. 526
A Dentist's Surroundings.............................. 526

Are Rhubarb Leaves Poisonous? ..... 527
Anæsthesia as Produced by Schleich, of Berlin ..... 562
An Apparatus for Chloroform-Oxygen Anæsthesia ..... 565
Air-Chambers ..... 575
As an Obtundant ..... 576
Bridge-Work ..... 71
Bleaching Teeth by Electricity ..... 101
Beaded Enclosures in Vulcanite Dentures ..... 285
Bleaching Teeth ..... 336
Borine ..... 430
Borine ..... 478
Beeswax in Canals ..... 479
Bicycling for Women ..... 570
Copper Amalgam ..... 46
Combination of Cohesive and Non-Cohesive Gold in Filling, 79
Carving of Block-Teeth ..... 116
Chronic Alveolar Abscess with Complications ..... 286
Cyst of the Antrum or Highmore ..... 288
Cobalt as a Pulp Destroyer ..... 333
Cork Wedges for Separating Teeth ..... 334
Cleft Palates—Cases in Practice ..... 369
Caries of Jaw From Impacted Wisdom Tooth ..... 420
Cocaine ..... 444
Circumcision ..... 476
Character in Teeth ..... 479
Cleaning Teeth ..... 523
Coal Oil in the Laboratory ..... 527
Changes in Nerve-Cells ..... 527
Dentistry and General Medicine ..... 5
Dental Surgery ..... 40
"Dental Medicine" ..... 45
Dr. Cunningham on Tooth Powders ..... 48
Death From Nitrous-Oxide Gas ..... 62
Dental Dots ..... 93
Decay of Teeth Coincident with Advanced Civilization ..... 95
Dead Teeth ..... 144
Dead Teeth ..... 157
Decay of the Teeth ..... 189
Digestion ..... 191
Decayed Teeth ..... 232
Disinfectants ..... 236
Duties of Dentist to Patient—Duties of Patient to Dentist ... 263
Dr. H. A. Wallace ..... 335
Disease of the Antrum of Highmore from a Medical Standpoint ..... 349
Dental Jurisprudence—Infection ..... 373
Dr. Thomas H. Chandler ..... 375

Disturbances Caused by Diseases of the Teeth .............. 383
Dr. Paine's Local Anesthetic ...... ........................ 399
Dr. L. P. Haskell on Dental Prosthesis ...................... 406
Died in a Dentist's Chair ........... ..................... 418
Dr. James E. Garretson ... ............................... 422
Dentist's Gold ......... ........... ................... 430
Died Under Gas ........... .. ........ ................... 464
Dental Enactments ..... ........ ........................ 466
Dental Faculties in Medical Schools ....................... 468
Death Due to Dentistry—A Woman's Fatal Bleeding After Sixteen Teeth Pulled ............................... 474
Dental Science Then and Now ........ .................. 481
Dr. Gorgas' Experience with Borine .... .... ........... 528
"Doc." ....................................................... 576
Extracts from Essays on Chinese Dentistry ............... 24
Erosion ......................................................... 188
Extracting an Abscessed Tooth ........................... 190
Epulis ............ ........................................ 320
Electrolysis in Arresting Erosion ............ ............ 336
Electrolysis for the Surgical Treatment of Strictures ........ 447
Extracting Teeth ....... .. .. ............................ 461
Fifty Years in the Laboratory; or The Evolution of the Dental Laboratory as I have seen it ...................... 145
Filling Pulpless Teeth with Fistulous Opening .. ............ 182
Friction-Massage with the Fingers .......................... 183
Formula for Pulp Devitalization ............................ 187
Filling Pulpless Teeth with Fistulous Opening ............. 231
For Hemorrhage ......... .... .............................. 240
Fitting Crowns .... ....................................... 285
For Setting Crowns ......... ............................. 286
Fracture of the Superior Maxilla .......................... 357
From German Dental Journals ... ......................... 415
Filling Children's Teeth .................................. 431
Furred Tongue ..... ....................................... 449
Glass Filling, Jacket Crowns, Amalgams, Gold Caps, Bridges, etc.—A Scrap of Their History ........................ 205
Gold and Porcelain Crowns ................................ 232
Good and Bad Teeth ..... ................................ 470
How to Get Clean Joints ................................... 94
How to Vulcanize Rubber Plates Between Metallic Surfaces, 114
Higher Dental Ethics ...................................... 218
Hygiene of the Eyes .. .................................... 235
How to Arrest a Boil, Carbuncle, or Malignant Pustule ..... 236
How to Vulcanize Rubber Plates Between Metallic Surfaces, 275
Hypnotism in Surgery ..................................... 282
Habit Spasms ............ ................................ 320
Harvard Odontological Society ............. ............ 529

Hemorrhage........ .............................. 274
Hypnotism in Surgery ... ........................... 574
Infected Root Canals.............. ...... ...... 235
Inflammation.......................................... 288
Is Oxygen a Simple Body ?............ .. ...... ...... 328
Infected Root Canals.................................. 334
Iron and the Teeth.............................. ... 475
Infiltration Anesthesia................................ 521
Illinois State Dental Society................... ........ 568
Killing an Abscess............... ...................... 89
Lining Rubber Plate with Aluminum...................... 186
Longevity.......... ................................ 546
Medical Men and Dentistry.............................. 38
Medicine and Dentistry....... ......................... 233
Metallurgy............................................ 249
Malignant Tumors of the Mouth .......... ............. 344
Men That Should Not be Tolerated in the Dental Profession, 431
Mammoth Teeth Unearthed............................... 472
Medical and Dental State Boards ........................ 572
North-western University Dental School.................. 85
Nucleln ........................................ ........... 164
New Local Anæsthetic.................................. 233
National Association of Dental Examiners................. 277
New Anæsthetic Mixture................................ 335
National Association of Dental Faculties.................. 364
New Method of General Narcosis......................... 575
Oxyphosphate of Zinc as a Filling Material—Its Abuse...... 21
Objections to Amalgam................................. 69
Odontological Society of Chicago........... ............ 137
Our Institutions........... ........................... 282
Obtunding Sensitive Dentine.............................. 287
Occlusion and Mastication............................. 358
Odontographic Society of Chicoge....................... 468
Preventive Dentistry...................................... 10
Program of the Second Union Meeting of the Washington City Dental Society and the Maryland State Dental Association, at the Royal Arcanum Hall, Baltimore, Md ... 41
Pyorrhœa Alveolaris................................... 55
Pericementitis Producing Abscess....... .. .............. 73
Physicians on the Canadian Border ...................... 140
Protecting Pulps......... .............................. 185
Plastics as a Bar to Bacteria........................... 258
Porcelain Faced Bicuspid Crowns.... .. ................ 283
Pulp Capping................................ ... 586
Pauperism............... ........................... 287
Physical Education..... ................ ............. 289
Peroxide of Hydrogen....... ............................ 329

Pulp Protection..... .................................... 330
Pyorrhea Alveolaris.......................................... 385
Pulp-Capping in Deciduous Teeth........................ 432
Poisoning by Lysol........... ............................. 479
Pacific Coast Dental Congress............................ 527
Proposed Maryland Dental Law............................ 568
Quackery Defined ............................................ 230
Rubefacients and Vesicants................................ 121
Root Canals..................................................... 191
Regulating ................................................... ... 311
Resetting or Remodeling a Rubber Plate ................. 335
Rules for the Setting of Oxy-Phosphate Cement Fillings.... 480
Some Queer Questions Regarding the Teeth.... ... ....... 48
Some Remarks on the Treatment of Inflamed Pulp........ 49
Southern Dental Association.............................. 86
Swallowed Plates.............................................. 87
Simple and Complicated Regulating Mechanisms; Æsthetic Mechanisms; Proper and Improper Anchorage.......... 111
Systemic Dissemination of Tuberculosis From a Patch of Lupus Vulgaris........................................ 281
Sixth-Year Molars.............................................. 314
Smooth Models ................................................ 336
Spirits of Camphor............................................ 336
Sir John Tomes........................................... ... 377
Systemic Medication for the Relief of Sensitive Dentine.... 403
Strange Case of a Degenerating Human Body.............. 429
Stoned Burs..................................................... 478
Systemic Treatment by Dentists........................ ... 518
Spitting in Public Places ................................. 520
Sterilization of Dental Instruments........................ 525
Salol Topically.................................................. 525
Salt.... ......................................................... 526
Surgery Without Pain..... ................................. 539
Some Thoughts Touching Excess of Mineral or Calcareous Matter in the System.................................. 540
The Dentist, His Relations to His Patient and to His Brother Practitioners...................................... 1
The Horace Wells Permanent Memorial.................... 9
The Preparation of Roots for Crowning and Gold Crowns.. 29
The Real Value of the Medicinal Peroxide of Hydrogen Preparations Found in the Market...... ............... 35
The Necessity of a Higher Estimation of Tooth and Mouth Hygiene....................................... ...... 47
Treatment of Broken Impressions........................... 96
The Dental Laboratory........................................ 103
The Administration of Chloroform......................... 141
The Influence of Pregnancy on Caries..................... 142

The Sanitary Sin........................................ 143
The Successful Treatment of Cleft Palate................. 154
The Structure of the Maxillary Bones..................... 175
The Dental Filling....................................... 181
Teeth of Our School Children............................. 182
Transmission of Cholera by the House Fly................. 184
The Preventi.e Treatment of Seasickness.................. 184
The Application of the Rubber Dam........................ 185
Tortuous Canals.......................................... 188
Thinking with the Finger Tips............................ 188
The Adulteration of Drugs................................ 193
Treating Nausea Caused by Taking Impressions............. 230
To Prepare Cavities...................................... 237
The Treatment of Epithelioma............................. 238
The Scientific Spirit and the Ethics of Dental Practice...... 241
Treatment of Asiatic Cholera............................. 250
The Importance of Prosthetic Dentistry................... 284
Transmission of Cholera by the House Fly................. 331
The First Work of the Dentist............................ 322
Treatment of Chloroform Asphyxia......................... 333
To Deodorize Iodoform.................................... 336
The Profession and Our Dental Colleges................... 337
The Tincture of the Chloride of Iron..................... 360
The Care of Children's Teeth............................. 380
To Open Up a Blind Abscess............................... 384
To the Members of the Maryland State Dental Association
and the Washington City Dental Society................... 426
The Anesthetic and Asphyxiating Effects of Nitrous-Oxid
Inhalation............................................... 452
To Lessen the Pain from Arsenic.......................... 480
The Origin of Salivary Calculus.......................... 497
To Secure Rubber Dam..................................... 523
To Raise the Standard of Medical Education............... 524
The Warm Bath in Insomnia................................ 528
The Origin of Salivary Calculus.......................... 554
Vitality Conditioned..................................... 47
Vulcanizing Between Metal................................ 186
Varnishing Cavities...................................... 332
Varnishing Cavities...................................... 432
What Dentists are Eligible to Membership in the American
Medical Association...................................... 86
What Dr. T. W. Brophy Says............................... 96
Will the Too Prevalent Use of Plastics in Filling Teeth
Work Injury to Operative Dentistry....................... 129
What Shall we do with the Temporary Teeth................ 306
Why Will Not Fire Burn Brightly When the Sun Shines
Upon It?................................................. 327
Wedging Teeth with Rubber................................ 330
What Dr. Garret Says of Pyorrhœa Alveolaris.............. 335

### To Readers and Correspondents.

Communications intended for publication, and exchange journals and papers, should be addressed to the Editor of The American Journal of Dental Science, No. 845 N. Eutaw St. Baltimore, Md. All letters relating to the business of the Journal, or containing cash remittances, to be directed to Snowden & Cowman Mfg. Co., 9 W. Fayette Street, Baltimore, Md. Articles for publication should be received by the Editor at least two weeks previous to the first of the month on which the number in which it is designed for them to appear, is issued.

Dental Surgery. - - - - - - - - - - - 40

Program of the Second Union Meeting of the Washington City Dental Society and the Maryland State Dental Association, at the Royal Arcanum Hall, Baltimore, Md. - - - - 41

### MONTHLY SUMMARY.

"Dental Medicine." - - - - - - - - 45

A Note on Capping a Tooth Pulp. - - - - - - 46

Copper Amalgam. - - - - - - - - - - 46

The Necessity of a Higher Estimation of Tooth and Mouth Hygiene. 47

Vitality Conditioned. - - - - - - - - - 47

Some Queer Questions Regarding the Teeth. - - - - - 48

Dr. Cunningham on Tooth Powders. - - - - - - 48

☞ The American Journal of Dental Science will contain fifty pages of reading matter in each number, making a volume of about Six Hundred pages,

EXCLUSIVE OF ADVERTISEMENTS

